# A Case of Bacterial Keratitis in a Patient Using Orthokeratology and Soft Contact Lenses

**DOI:** 10.7759/cureus.68717

**Published:** 2024-09-05

**Authors:** Yota Kikuchi, Hiroshi Toshida, Junji Ono

**Affiliations:** 1 Ophthalmology, Juntendo University Shizuoka Hospital, Shizuoka, JPN; 2 Ophthalmology, Ono Eye Clinic, Shizuoka, JPN

**Keywords:** complication, corneal ulcer, high myopia, microbial keratitis, orthokeratology, pseudomonas aeruginosa, rigid gas permeable contact lens, soft contact lens

## Abstract

A 63-year-old male with high myopia developed sudden visual loss, eyelid swelling, eye pain, discharge, and tearing in his left eye while wearing soft contact lenses (CLs) during the day and orthokeratology lenses at night. At the initial visit, his corrected visual acuity in the left eye was 20/1000, with a ring-shaped ulcer in the central cornea, corneal infiltration across the entire cornea, and conjunctival hyperemia. *Pseudomonas aeruginosa* was detected from corneal scrapings, and after antibiotic treatment, the ulcer healed with corneal opacity remaining.

On the 60th day, corrected visual acuity of 20/20 was achieved with rigid gas-permeable CLs. To prevent CL-related ocular complications, eye care professionals must carefully evaluate the suitability of all CLs, including orthokeratology.

## Introduction

Orthokeratology was approved for refractive correction by the US Food and Drug Administration in 2002 and has since become widespread globally [1-3]. In Japan, it was approved by the Ministry of Health, Labour, and Welfare in 2006. Subsequently, a group of ophthalmic specialists in the Japanese Ophthalmological Society and Japan Contact Lens Society created the Orthokeratology Guidelines. The guidelines were revised in 2017, but the criteria for refractive correction remained the same, i.e., myopia up to -4D and corneal astigmatism up to -1.5D [4]. In practice, some prescriptions exceed these criteria; however, significantly exceeding them is known to not only make full correction unlikely but also to increase the risk of ocular complications [5].

We report a case of a patient with high myopia exceeding -13D for whom a non-ophthalmic facility prescribed overnight orthokeratology lenses not approved in Japan to compensate for insufficient correction by regular frequent replacement soft contact lenses (FRSCLs) that he had been wearing throughout the day. Several years later, the patient developed bacterial keratitis.

## Case presentation

The patient was a 63-year-old man. Because of insufficient correction with -10D Acuvue Oasys® FRSCLs (Johnson & Johnson Vision Care, Inc., Jacksonville, Florida, USA) alone, 10 years ago he began using orthokeratology lenses prescribed by a non-ophthalmic facility. Throughout the day, he wore FRSCLs and, at night, orthokeratology lenses. The types and usage of the lenses are shown in Table 1.

**Table 1:** Summary of lens types, usage, prescription, and notable details FRSCL: frequent replacement soft contact lens

Lens type	FRSCL	Orthokeratology lens
Lens brand name	Acuvue® Oasys® (Johnson & Johnson)	Unknon, unapproved lenses in Japan
Base curve	8.80mm	Unknown
Diopter	−10.0D	Unknown
Diameter	14.0mm	Unknown
Wearing schedule	Daily	Overnight
Lens care solution	Unknown	Unknown
Purchase location	Initially prescribed by a local eye clinic; self-purchased online for the past 4 years	Prescribed by a non-ophthalmologist
Note	Prescribed -10.0D lenses (though product available up to -12.0D)	Used to partially correct undercorrection with FRSCL

Over the 10 years, the patient had been wearing orthokeratology lenses of an unspecified brand, which were unapproved in Japan and had replaced the lenses with new ones approximately every two years. At the time of referral to our facility, he had been using the current orthokeratology lenses for one and a half years. The care products used were unknown. Thus, for the past 10 years, the patient has been wearing some type of contact lens for almost 24 hours a day, 365 days a year.

Two days before visiting the Ono Eye Clinic, in Shizuoka, Japan, the patient experienced a sudden decrease in visual acuity in his left eye, eyelid swelling, eye pain and discharge, and tearing. He was diagnosed with infectious keratitis and referred to the Department of Ophthalmology at Juntendo University Shizuoka Hospital, Shizuoka, Japan, on the same day for further examination and treatment. His past medical history included rheumatoid arthritis, for which he was taking oral steroids. At the initial visit, his right eye had an uncorrected visual acuity (UCVA) of 20/250 and a best corrected visual acuity (BCVA) of 20/20, while the left eye had a UCVA of 20/1000 and was uncorrectable. Objective refraction and keratometric values measured by ARK-1A (NIDEK, Aichi, Japan) are displayed in Table 2. Originally, the refraction values were nearly identical in both eyes, as reported by the patient.

**Table 2:** The refraction and keratometric values at the first visit SPH: spherical power; CYL: cylindrical power; D: diopters; deg: degrees; R1: radius 1; R2: radius 2; Ave: the average of R1 and R2 values

	Right eye			Left eye		
Refraction value	SPH (D)	CYL (D)	Axis (°)	SPH	CYL	Axis (°)
	-13.75D	-2.25D	27	error	error	error
Keratometric value	mm	D	deg (°)	mm	D	deg (°)
R1	7.58	44.50	128	7.94	42.50	109
R2	7.49	45.00	38	7.70	43.75	19
AVE	7.54	44.75		7.82	43.25	
CYL		-0.50	128		-1.25	109

**Figure 1:**
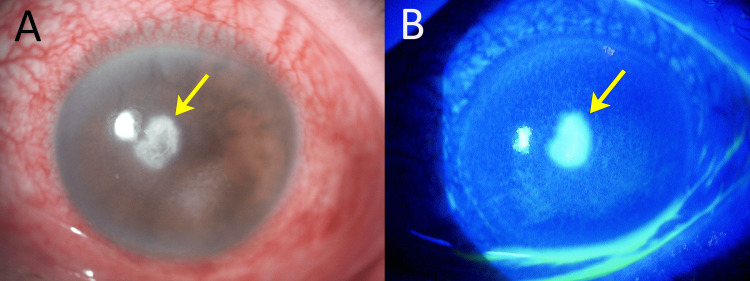
Ocular findings at the initial visit Slitlamp microscopic images of the left eye (A), stained with fluorescein (B) at the first visit. (A) Microscopic image of the left eye: A small, well-defined ring-shaped abscess was observed in the central cornea. The cornea exhibited a generally frosted appearance, accompanied by conjunctival hyperemia. (B) Fluorescein dye staining: Fluorescein staining revealed areas corresponding to the corneal ulcer. Note: The arrows indicate the location of the corneal ulcer in both images.

Upon examination, the right eye showed no significant changes from the anterior segment to the fundus; however, the left eye showed conjunctival hyperemia, a ring-shaped corneal abscess in the central cornea, and corneal infiltration extending throughout the cornea (Figure 1), and the fundus was not visible.

The corneal endothelial cell density, coefficient of variation (CV), and hexagonality (HEX) measured by Noncon ROBO (Conan Medical, Hyogo, Japan) were 2313 cells/mm², 42%, and 48%, respectively, in the right eye. On the other hand, in the left eye, due to corneal opacity, the images were unclear, and measurements were likely inaccurate.

Based on these findings, a *Pseudomonas aeruginosa *infection was suspected. On the same day, the patient was hospitalized, and antibacterial treatment was started with frequent administration of levofloxacin 1.5% eye drops (Cravit®, Santen Pharmaceutical Co., Ltd., Osaka, Japan), cefmenoxime hydrochloride 0.5% eye drops (Bestron®, Senju Pharmaceutical Co., Ltd., Osaka, Japan), gentamicin 0.3% eye drops (Gentamicin, Nitto Medic Co., Ltd., Toyama, Japan) eye drops to the left eye, and ofloxacin 0.3% eye ointment (Tarivid®, Santen Pharmaceutical Co., Ltd., Osaka, Japan) was administered once before bedtime. In addition, imipenem/cilastatin IV infusion 0.5 g (Tienam®, MSD, Tokyo, Japan) was administered twice daily until the bacterial culture results were confirmed. Five days later, *P. aeruginosa* was detected from both the corneal scrapings and conjunctival sac scrapings, including eye discharge. The sensitivity to the administered antibiotics was confirmed, as shown in Table 3.

**Table 3:** Antimicrobial susceptibility profile of corneal scraping Similar results of *Pseudomonas aeruginosa* were observed in the conjunctival sac scrapings. Based on these results, sensitivity to levofloxacin was confirmed, so the antibacterial eye drops were limited to levofloxacin only. Some fields under the 'Evaluation of Antibacterial Susceptibility' section were left blank. MIC: minimum inhibitory concentration; S: sensitive; I: intermittent; R: resistant

Name of antimicrobial	MIC	Evaluation of antibacterial susceptibility
Piperacillin	<=4	S
Ampicillin/sulbactam	>=32	R
Tazobactam/Piperacillin	<=4	S
Ceftazidime	4	S
Cefepime	4	S
Azithromycin	8	S
Imipenem/Cilastatin	2	S
Meropenem	0.25	S
Amikacin	<=4	S
Gentamicin	<=2	S
Tobramycin	<=2	S
Minocycline	>=16	R
Clindamycin	2	
Levofloxacin	1	S
Ciprofloxacin	<=0.5	S
Sulfamethoxazole/Trimethoprim	38	R
Ceftolozane/Tazobactam	<=1	S

**Figure 2:**
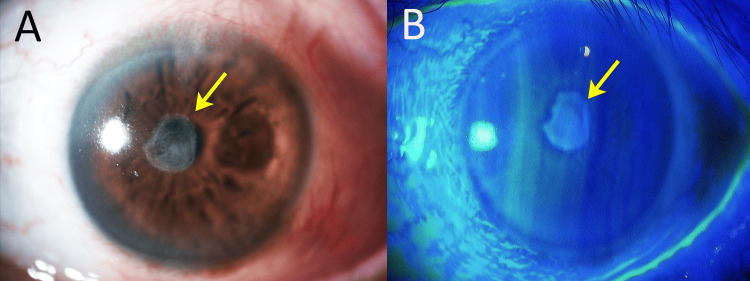
Ocular findings in the left eye at discharge (A) Both conjunctival hyperemia and subepithelial opacity had decreased. (B) The fluorescein staining image showed a resolution of corneal epithelial damage. Uncorrected visual acuity was 20/300 in the left eye. Best-corrected visual acuity was 20/125 with -9.00D Sphere. Note: The arrows indicate the location of the corneal ulcer in both images.

All medications except for levofloxacin 1.5% eye drops and ofloxacin 0.3% eye ointment were discontinued. At the two-week mark from the initial visit, although corneal opacification remained, the ulcer had healed with scarring (Figure 2), and the patient was discharged.

**Figure 3:**
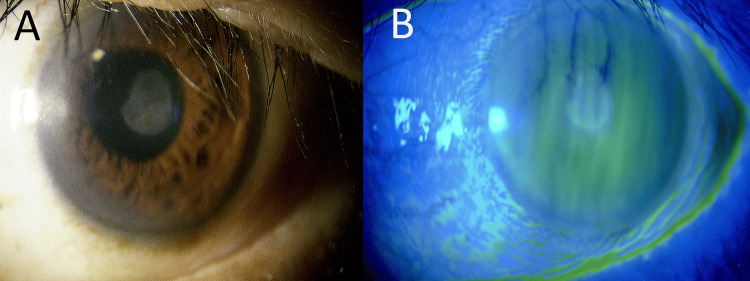
Clinical findings at two months after the initial visit (A) The conjunctival hyperemia in the left eye had resolved, and the subepithelial corneal opacity had further faded. (B) No fluorescein staining was observed. Although the infection was suppressed and the inflammation subsided, irregular corneal astigmatism caused by the corneal ulcer persisted. Uncorrected visual acuity was 20/300 in the left eye, and best-corrected visual acuity was 20/70 with -9.00D Sphere.

**Figure 4:**
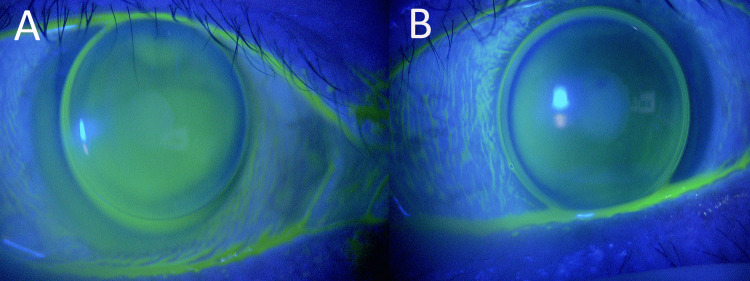
RGP lens fitting Two months after the initial visit, rigid gas-permeable (RGP) contact lenses were then fitted in both eyes to correct the irregular astigmatism caused by the corneal ulcer and to address high myopia. The RGP lenses used were AS LUNA (Seed Co., Ltd., Tokyo, Japan). The lens parameters were 7.60/-10.75/9.2 for the right eye (A) and 7.70/-9.00/9.2 for the left eye (B), respectively.

At discharge, the BCVA of the left eye was 20/125. The corneal endothelial cell density, CT, and HEX in the left eye were measurable and were 2000 cells/mm², 41%, and 45%, respectively. The hyaluronic acid 0.1% eye drops (Hyalein®, Santen Pharmaceutical Co., Ltd., Osaka, Japan) and bromfenac sodium hydrate 0.1% eye drops (Bronuck®, Senju Pharmaceutical Co., Ltd., Osaka, Japan) were added. One week later, fluorometholone 0.1% eye drops (Flumetholon®, Santen Pharmaceutical Co., Ltd., Osaka, Japan) was added. Two months later, scar healing was confirmed (Figure 3) and a BCVA of 20/20 was achieved with rigid gas permeable (RGP) contact lenses, and the patient began wearing RGP lenses in both eyes (Figure 4). The RGP lenses prescribed were AS-LUNA (Seed Co., Ltd., Tokyo, Japan), and the lens care solution used was One O Care (Aime K.K., Yokohama, Japan).

**Figure 5:**
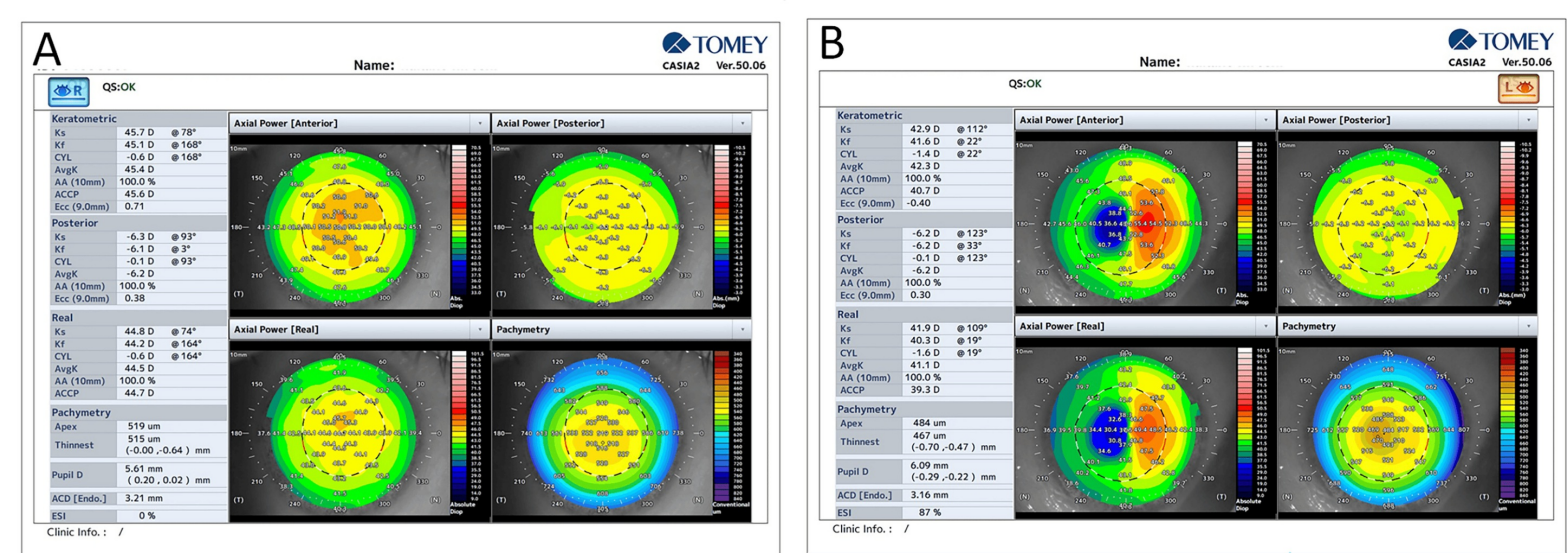
Corneal topography (A) Corneal topography of the right eye appeared nearly normal. (B) In contrast, the left eye exhibited irregular astigmatism, and asymmetrical components were prominent.

Corneal topography analysis using CASIA2 (Tomey Corporation, Aichi, Japan) showed that the right eye was nearly normal, while the left eye exhibited irregular corneal astigmatism (Figure 5).

At seven months after the patient started using RGP lenses, no recurrence of the infection has occurred and good BCVA has been maintained. Objective refraction and keratometric values at the last visit are displayed in Table 4.

**Table 4:** The refraction and keratometric values at the last visit SPH: spherical power; CYL: cylindrical power; D: diopters; deg: degrees; R1: radius 1; R2: radius 2; Ave: the average of R1 and R2 values

	Right eye			Left eye		
Refraction value	SPH (D)	CYL (D)	Axis (°)	SPH	CYL	Axis (°)
	-13.00D	-0.75D	22	-11.50D	-0.75D	140
Keratometric value	mm	D	deg (°)	mm	D	deg (°)
R1	7.52	45.00	164	7.77	43.50	81
R2	7.44	45.25	74	7.62	44.25	171
AVE	7.48	45.00		7.70	43.75	
CYL		-0.25	164		-0.75	81

The refraction and keratometric values in the left eye, which were unmeasurable at the initial visit, were successfully measured. At the final visit, nine months after the first visit, the refraction value in the left eye had decreased compared to that in the right eye.

## Discussion

The criteria for using orthokeratology lenses vary slightly by country. While many countries target myopia up to -6D, in Japan, their use is limited to a spherical degree of up to -4D [4]. The application criteria of the latest orthokeratology lens guidelines in Japan are shown in Table 5.

**Table 5:** Excerpt of indications from the Japanese Orthokeratology Guidelines This is an excerpt from the second edition of the Orthokeratology Guidelines, revised in 2017. Given the uncertainty regarding the long-term prognosis of refractive correction with Orthokeratology and the potential changes it may cause to a normal cornea, careful selection of candidates is necessary. Original version [4]* (in Japanese)*.

1. Age	
	As a general principle, the patient should be 20 years or older, based on the idea that sufficient judgment and consent can be obtained from the patient without requiring the involvement of a guardian. Prescribing Ortho-K for those under 20 years of age should be done with caution.
2.Target	
	Candidates should have stable refractive errors such as myopia or astigmatism.
3. Degree of refractive correction	
	1) Myopia correction is generally limited to up to -4.00D. Astigmatism correction is limited to up to -1.50D. In cases of clear against-the-rule astigmatism or oblique astigmatism, careful consideration should be given before prescribing.
	2) The central corneal refractive power should be between 39.00D and 48.00D.
	3) The goal is to avoid overcorrection after treatment.
4. The patient should have healthy eyes without any eye diseases and meet the following criteria	
	1) No significant fluorescein staining of the corneal and conjunctival surfaces, and a Schirmer I test result of 5 mm or more within five minutes.
	2) Corneal endothelial cell density should be 2,000 cells/mm² or more.

In the present case, the spherical degree was outside the range specified by the orthokeratology lens guidelines (Table 5). Additionally, the orthokeratology lenses used by the patient were not approved in Japan. The patient had rheumatoid arthritis and was taking systemic steroids, which suppressed his immune system, making even the use of standard SCLs risky [5].

The combined use of orthokeratology lenses and another CL is not recommended. Some SCLs now provide degrees of correction exceeding -10D, but RGP lenses could have been made for the patient from the start. The patient was not informed by either the store whether he purchased the FRSCLs or the clinic handling the orthokeratology lenses that correction could be achieved with a single type of lens. Even if the prescribing doctor or facility was not able to prescribe contact lenses that provided the necessary refractive correction, they should have explained the situation to the patient and referred him to a facility that could prescribe more suitable contact lenses.

Keratitis due to *P. aeruginosa* or Acanthamoeba is one of the severe ocular complications associated with CLs and orthokeratology lens use [5-12]. And in severe cases, it can lead to corneal perforation or endophthalmitis [13,14]. There have been reports showing different trends in the changes of conjunctival sac bacterial flora after wearing CLs [9,15], especially SCLs and orthokeratology lenses [9]. According to this report, the changes were more significant with orthokeratology lenses, although there were no cases where both types were used simultaneously. Thus, it is possible that changes in the conjunctival sac bacterial flora occurred due to the wearing of SCLs and orthokeratology lenses.

In the present case, because of the corneal findings at the initial visit and CL use history, we immediately suspected infectious keratitis due to *P. aeruginosa* and started treatment accordingly. The initial treatment was effective and resulted in relatively early infection control. However, as a sequela of bacterial keratitis, corneal scarring, mild subepithelial opacity, and irregular corneal astigmatism remained. After healing, RGP lenses were deemed appropriate for correction of post-ulcer irregular astigmatism and high myopia. Because orthokeratology lenses are made of oxygen-permeable material, the transition to wearing all-day RGP lenses was easy for the patient. The RGP lenses were prescribed and provided detailed instructions on the risks of wearing contact lenses, precautions with RGP use, proper lens care, and how to handle ocular complications. Since then, no ocular complications have occurred.

A notable, though silent, ocular complication in this case was a decrease in corneal endothelial cell density. The patient had lower corneal endothelial cell density than the average values for a Japanese person of his age [16]. As mentioned, he wore FRSCLs throughout the day and orthokeratology lenses at night for about 10 years, almost 24 hours a day. Even though both lenses are considered highly oxygen permeable, such prolonged use, if accompanied by lens degradation, dirt, poor fitting, or reduced tear exchange rates leading to a lack of oxygen supply, could have impacted corneal endothelial cells. Although the patient now only wears RGP lenses during the day and no contact lenses at night, his corneal endothelial cell condition requires continuous monitoring.

## Conclusions

We encountered a case of infectious keratitis in a patient who had worn FRSCLs during the day and orthokeratology lenses at night for approximately 10 years. We believe that wearing contact lenses for nearly 24 hours a day was a significant risk factor for the development of corneal infection. Additionally, there was a decrease in corneal endothelial cell density.

After treating the infectious keratitis and its sequelae, RGP lenses were determined to be suitable for correcting both the residual irregular astigmatism following the corneal ulcer and the patient's high myopia. Eye care professionals who prescribe contact lenses must possess a thorough understanding of these devices and ensure they provide patients with the most appropriate prescription.
